# Lung ultrasound for causal diagnosis of shock (FALLS-protocol), a tool helping to guide fluid therapy while approaching fluid tolerance. Some comments on its accuracy

**DOI:** 10.1186/s13613-024-01329-8

**Published:** 2024-06-14

**Authors:** Daniel A. Lichtenstein, Stéphane Bar

**Affiliations:** 1 Medical Intensive Care Unit of Hospital Ambroise Paré, Paris-Saclay University, 9 avenue Charles de Gaulle, Boulogne, France; 2 Department of Anaesthesiology and Critical Care Medicine, Amiens University Hospital, 1, Rond-Point du Professeur Christian Cabrol, 80000 Amiens, France

The FALLS-protocol (fluid administration limited by lung sonography) is an ultrasound approach done at admission, facilitating causal diagnosis of acute circulatory failures with no obvious cause through seven steps. Whereas the six first steps are validated, the final step (appearance of B-lines under fluid therapy) has been only recently assessed [1]. The present article goes further to explain the relevance of this preliminary work in order to reposition this seventh step in the progression of the FALLS-protocol.

Very schematically, following Weil’s classification, the FALLS-protocol first uses echocardiography for ruling out pericardial tamponade (Step-1) then right ventricle enlargement suggesting first of all pulmonary embolism (Step-2). It then assesses lung sliding, ruling out pneumothorax (Step-3), and therefore obstructive shock.

Step-4 searches for the B-profile of the BLUE-protocol, highly correlated with acute hemodynamic pulmonary edema (AHPE). With patients in shock, AHPE suggests left cardiogenic shock. Note that the B-profile is not just “B-lines”, as often thought, but more than two B-lines between two ribs, symmetrically distributed anteriorly, and associated with lung-sliding [2]. The B-profile is a direct sign of AHPE. Classical tools (Doppler-echocardiography etc.) will then search for the cause of this left cardiogenic shock. Absence of B-profile rules out AHPE (that is, logically, left cardiogenic shock).

Step-5 is applied when the A-profile is seen (anterior A-lines mostly, with lung sliding). The B-line appears from 18-mm-Hg of pulmonary artery occlusion pressure (PAOP) [3]. A-lines, indicating non-elevated PAOP, are a logical invitation to administer fluids. The idea is to partially treat both remaining causes of shock (hypovolemic, distributive), while promptly detecting the transformation from A-lines to B-lines under fluid therapy, a change called the FALLS-profile (Step-7).

Step-6 is meanwhile an ultrasound search for any source of hypovolemia (e.g., bleeding) or sepsis (e.g., pneumonia, generating four profiles [2]). Step-6 is called Round-FALLS-protocol. When Step-6 is positive (allowing the diagnosis), the fluid therapy can be managed using traditional rules, or continued until a FALLS-profile occurs (with the idea of correcting the hypovolemic part). Each time Step-6 is negative, Step-7 is a logical option. Regular views of the lung and clinical metrics are done. If a FALLS-profile occurs, without clinical improvement, fluid administration is discontinued: in hypovolemic shock, the circulation would logically improve before fluids begin to invade the lung, so hypovolemic shock becomes unlikely. The FALLS-profile therefore immediately indicates distributive shock (by default) usually *septic shock*. The FALLS-profile indicates a septal edema (infraclinical, biologically occult step of AHPE) [4, 5] just occuring. The FALLS-profile strongly suggests the specific moment for improving circulation by any other mean (introducing vasopressors e.g.).

The *raison d’être* of the FALLS-profile is to administer just the amount of fluid needed to generate the first B-lines. In theory, the FALLS-profile, an all-of-a-sudden phenomenon, may be generated by one additional *drop* of fluid.

The FALLS-protocol has a few real limitations (exceptional giant anterior bulla). The FALLS-profile cannot be generated if B-lines are initially present, schematically. The FALLS-protocol does not intend to oversimplify the rules of expert hemodynamic assessment, a huge field. It is not yet devoted to assessing daily needs in fluids in a ventilated patient. Space lacks for dealing with many subtleties (e.g., lung sepsis can generate right ventricle enlargement, causing pneumonia to be suspected at Step-2, and inviting caution before considering fluid therapy).

The article [1] is the first to our knowledge providing data concerning the FALLS-profile, which was compared to a tool assessing fluid unresponsiveness in the operating room. The sensitivity is 80%, and the specificity 57%. This imperfect correlation was fully expected (see below). We have data, however, which provide a basis for discussion. What can we conclude? The tested sign was the FALLS-profile, the disease fluid unresponsiveness, the reference esophageal Doppler. Half of these patients (43%) had the sign and not the disease: they developed the FALLS-profile, while the current approach suggested giving additional fluids. This is the opportune time to ask: what is the value of the reference test? Was esophageal Doppler *really* validated? Was any hemodynamic tool, including elegant and logical concepts, such as fluid responsiveness, *really* validated? Our tentative conclusion is that trying to validate a new approach, using references which have not been validated, generates the logical risk of obtaining suboptimal results. Other studies can be done with other tools, but the quandary will not likely disappear.

We sought alternative explanations. Development of pneumonia during the perioperative period is unlikely, the follow-up showing a low percentage of pneumonia (8%). Peri-operative atelectases don’t generate B-lines, especially anteriorly. Suddenly developing interstitial pulmonary fibrosis precisely during surgery is not a serious explanation. Therefore, these B-lines likely appear as hemodynamic B-lines.

Readers will note that 43% of patients in the study left the operating room with potential AHPE [1]. This may appear worrisome, just because the term “pulmonary” edema is curiously assimilated in most minds as “alveolar” edema. But it can also be *interstitial* edema, more especially *septal* edema first (FALLS-profile). The study [1] did not see 43% of patients in overt AHPE in the postoperative stage: only 2%. This shows how silent the FALLS-profile is. Interstitial (hemodynamic) edema invariably *precedes* alveolar edema [4]. The study [1] provides one further validation that interstitial AHPE is a silent development, with moderate or no effect on gas exchanges.

We already have numerous tools for analyzing needs for fluid administration (Doppler echocardiography, SVV, PPV, ITV, splanchnic venous Doppler, PLR etc.). We now have one more. We simply highlight two points.

1. This approach, showing an asymptomatic disorder, may be of interest at times where fluid tolerance is increasingly considered.
2. It is, for once, based on an on–off pathophysiological change, therefore independent of numbers, whereas most other hemodynamic tools (which can and must be associated at any time), provide data based on continuous numeric values.

Simply for this ability of early recognition of extravascular lung water excess, one may envisage the FALLS-profile as another gold-standard, until validated proof of the contrary. Meanwhile, it may be associated (or compared) to current gold-standards. We therefore invite the Community to consider this potential alongside the traditional tools.

## Data Availability

Not applicable.

## Abbreviations

FALLS: Fluid administration limited by lung sonography
PAOP: Pulmonary artery occlusion pressure
mmHg: Millimeter of mercury
